# Some Observations and Cases, Shewing the Danger of Suppressing Accustomed Evacuations, or Rashly Interfering with Certain Periodical Affections of the System

**Published:** 1819-07-01

**Authors:** William Norman

**Affiliations:** Surgeon, Langport


### *Some Observations and Cases, shewing the Danger of suppressing accustomed Evacuations, or rashly interfering with certain periodical Affections of the System.*

By William Norman, Surgeon, Langport.

If peculiar events, passing within the immediate sphere of one's own observations, furnish criteria by which to estimate corresponding occurrences in Society at large, I have abundant reason for believing that many valuable lives continue to be endangered or destroyed by the pernicious practice of suddenly suppressing, or violently interrupting long-accustomed evacuations from, and affections of, the human system.

The subject of this remark is indubitably one of vital importance to the interests of mankind; and although the medical profession has been amply enlightened on this topic by the writings of several illustrious pathologists of our own times,* yet it is evident that its principles have not been sufficiently diffused, to awaken unsuspecting valetudinarians to a sense of the Proteian evils that so often and so insidiously surround them.

Gout, hæmorrhoids, erysipelas, rheumatism, eruptive diseases, ulcers, &c. are considered as unqualified and positive evils, unredeemed of their obnoxiousness by any attendant benefit. The heedless impatience for dismissing these, doubtless, severe visitations, prevents that calm contemplation of their causes and effects to which the beneficence of Nature entitles them, and which the welfare of the sufferer ought unquestionably to enjoin.

Medical science, in its present state, affords but few fixed rules or determinate principles to govern with infallible rectitude the tendencies of our minds, or to guide, with unerring precision, the dictates of our judgments; and our best practical deductions confessedly owe, in many instances, their origin and institution to hypothetical speculation alone. Therefore not only is every one at liberty to *entertain* his peculiar theories and opinions on whatever medical subjects happen to fall under his cognizance, but the cause of humanity, which it is his bounden duty to promote to the utmost of his ability, often requires that he should publicly *assert* them also. By so doing, investigation is excited, discussion elicited, contrarieties of sentiment displayed, and truth and science advanced—provided always that investigation be conducted in the "calm light of mild philosophy, and discrepancies argued in the dignified spirit of rational liberality."

Whilst I adduce these circumstances as an apology for obtruding myself on your notice, I disclaim every desire to provoke, through

* Vide the Works of Parry, Johnson, Thomas, Scudamore, Armstrong, and others.

the medium of your invaluable publication, unnecessary controversy, or to sully its pages with virulent disputation.

Without affecting, then, to propound my conclusions on a definite knowledge of the wise and mysterious laws of Nature, by which the structure and functions of the human machine are established, associated, and controlled; but, confiding their claim to public credit, on the testimony of long experience and attentive observation, corroborated by the authority of the ablest writers, I venture to suggest the opinion that Gout and the other peculiar states of system already referred to, are not virtually the opprobria of medicine, and the enemies of health, but, on the contrary, are spontaneous efforts of the constitution, to avert the more dangerous ascendancies of pre-accumulated and excessive dispositions to morbid action within it; and that by whatever means their sudden suppression or violent interruption be effected, notwithstanding that in their progress they should apparently exceed necessary limitations, these means will sooner or later be followed by the most baleful consequences.*

Minute pathological demonstration is not requisite to attest the validity of these positions; enough will be attained to establish their truth by a cursory view of the occasional states of system in a person who has been long accustomed to such periodical visitations, as those of gout, hæmorrhoids, or erysipelas. This view will shew the most important viscera of the body oppressed in their functions, and struggling with indigestion, flatulence, constipation, head-ache, giddiness, languor, ennui, and various other morbid phenomena. One or other of the conditions or diseases alluded to, now makes its appearance, and proceeds in regular progress to its termination — leaving the previous sufferer in the enjoyment of more ease and alacrity, both of mind and body, than he had for a long time before experienced. I beg leave to illustrate these positions by a few (out of many) cases in point, that have occurred in my own practice, or have otherwise come within my personal knowledge; pursuing the order of arrangement adopted in enumerating the conditions, or, as they are termed, diseases to which they are intended to refer.

Case I. A gentleman of fortune, then about 35 years of age, plethoric, but of temperate habits, had long been accustomed to attacks of gout in his lower extremities, and had occasionally applied cold water to the inflamed joints, during the paroxysms, without experiencing any ill effect from it. On one occasion, however, whilst labouring under an attack of gout in his feet, he plunged them into cold water; the inflammation quickly receded, and he became speechless. Reproduction of the gouty affection in his feet recovered his speech, and he did well.

Case II. An elderly gentleman, rather plethoric, somewhat

---

* Vide Medico-Chirurgical Journal for October 1818, p. 146.

disposed to convivial society, but yet not intemperate, and long accustomed to gout, was, about a year ago, suddenly relieved of a fit of the disease by taking the prescribed dose of "Reynolds's Specific." Apoplexy occurred on the following morning, from the effects of which he is not, I understand, at this time recovered.

Case III. A commercial gentleman had been, from his youth, periodically subject to hæmorrhoidal discharges, which, at a more advanced period of life, wholly forsook him. Within such an interval afterwards, as to lead to the suspicion that the affection might have resulted from the cessation of these long-accustomed evacuations, he was seized with severe disease of the thoracic viscera, and after having submitted to copious general bleedings, &c. during two or three years, he at length became dropsical, and died.*

Case IV. A young lady had been some years subject to hæmorrhoids, alternating with asthma. The discharges from the former becoming apparently excessive, or, in other words, becoming irksome to peculiar female habits, remedies were successfully resorted to for their abatement, if not for their entire suppression. A train of the most formidable cardiac and anomalous affections supervened, which, for a considerable time, not only baffled the remedial efforts that were employed to subdue them, but which placed the life of the patient in very imminent danger.

Case V. A farmer applied, by the advice of his neighbour, cold vinegar and water to an erysipelatous affection diffused over one of his legs. The inflammation of the leg speedily yielded to the cold applications, and was followed by inflammation of the stomach, from which he was with difficulty saved.

Case VI. A shoemaker, 40 years of age, thin, and of temperate habits, had been afflicted with acute rheumatism, at different times, for many years. During a severe attack of the disease in his knees, he applied cold water to them. Sudden recession of the

---

* My information relative to the hæmorrhoidal flux, and to the subsequent thoracic attack, together with the history of the treatment in this case, was voluntarily furnished by the patient himself, a few weeks only prior to his death. If his report was correct, (and from his great intelligence I have no reason to doubt it was) the conclusion above hazarded, as to the cause of the disease of the chest, may be considered as correct also. On that ground, therefore, it is much to be regretted, even as a practical omission, that steps were not taken to restore the hæmorrhoidal drain.

☞ The Editor, whose attention has long been directed to hæmorrhoidal affections, has met with many unequivocal cases of severe cerebral and thoracic, as well as other visceral disorders, resulting from the suppression of the hæmorrhoidal discharge. In his own practice he has also met with three distinct cases of pulmonary phthisis quickly coming on, after long established *fistulæ in ano* had been cured by a surgical operation. Ed.

inflammation occurred; an alarming affection of the chest ensued; and his life was, for a long time, despaired of.

This person, about five years afterwards, again had recourse to the cold water treatment for alleviation from (as he expressed himself) insupportable agony, under a similar attack of rheumatism. Recedence of inflammation from the knee took place, and was rapidly succeeded by inflammation of the brain, of which he died in about thirty-six hours.

Of the transformation of eruptive diseases into other diseases of different tendency, only one case, at present, occurs to my recollection; and as the treatment of that case is still under my observation, it were premature to notice it further in this place.

Case VII. A respectable widow woman, about 50 years of age, and of irreproachable habits of life, was attacked with violent symptoms of acute inflammation of the liver. General and topical bleedings* with other usual remedies were unavailingly resorted to. The disease resisted the most vigorous treatment, until it was discovered that a long-standing and profusely discharging ulcer of the heel had, during a temporary confinement to her house, a little while before, been nearly healed. The propriety of reproducing ulceration on the same part suggested itself; attempts to that end were made; they succeeded; and the patient's recovery uninterruptedly followed.

Although I have thus protested against the practice of suddenly suppressing, or rudely interrupting, certain actions and conditions of the human machine, and have designated such a course of proceeding as pernicious, I am, nevertheless, far from even questioning the propriety of attempting their removal, or modification, by the adoption of treatment that shall eradicate *constitutionally* the *causes* of their existence.

WILLIAM NORMAN.

Langport, 21 March, 1819.

---

* It is a humiliating circumstance to perceive that some medical practitioners of reputed talent and experience, in the nineteenth century, are denouncing *topical* bleeding as useless and unnecessary. If the understandings of such practitioners be not under the absolute dominion of prejudice and erroneous reasoning, to the total preclusion of conviction, an instructive lesson would be afforded them by a dispassionate reference to Johnson's "Influence of Tropical Climates," or, "The Atmosphere of the British Isles;" to Parry's "Elements of Pathology;" or to Yeats on "Hydrencephalus." Either of these valuable productions would clearly exhibit the fallacy of their doctrines, and would set their judgments right, on a highly important point of practice.

